# Antiviral prophylaxis in patients with solid tumours and haematological malignancies—update of the Guidelines of the Infectious Diseases Working Party (AGIHO) of the German Society for Hematology and Medical Oncology (DGHO)

**DOI:** 10.1007/s00277-015-2447-3

**Published:** 2015-07-21

**Authors:** Michael Sandherr, Marcus Hentrich, Marie von Lilienfeld-Toal, Gero Massenkeil, Silke Neumann, Olaf Penack, Lena Biehl, Oliver A. Cornely

**Affiliations:** Gemeinschaftspraxis für Hämatologie und Onkologie, Röntgenstr. 4, 82362 Weilheim, Germany; Klinik für Hämatologie, Onkologie und Palliativmedizin, Städtisches Klinikum Harlaching, München, Germany; Klinik für Innere Medizin II, Hämatologie und Internistische Onkologie, Universitätsklinikum Jena, Jena, Germany; Medizinischen Klinik II, Klinikum Gütersloh, Gütersloh, Germany; Ambulantes Onkologiezentrum Wolfsburg, Wolfsburg, Germany; Medizinische Klinik mit Schwerpunkt Hämatologie, Onkologie und Tumorimmunologie, Charité Universitäts medizin Berlin, Campus Virchow Klinikum, Berlin, Germany; Klinik I für Innere Medizin, Unklinik Köln, Köln, Germany; Deutsches Zentrum für Infektionsforschung (DZIF), partner site Bonn-Köln, Köln, Germany; Zentrum für Klinische Studien ZKS Köln, BMBF 01KN1106, Centrum für integrierte Onkologie CIO KölnBonn, Cologne Excellence Cluster on Cellular Stress Responses in Aging-Associated Diseases (CECAD), Medizinische Fakultät, Universität zu Köln, Köln, Germany

**Keywords:** Guideline, Antiviral prophylaxis, Hepatitis B, Cancer treatment

## Abstract

Reactivation of viral infections is common in patients with solid tumour or haematological malignancy. Incidence and severity depend on the extent of cellular immunosuppression. Antiviral prophylaxis may be effective to prevent viral reactivation. In 2006, the Infectious Diseases Working Party of German Society for Hematology and Medical Oncology (DGHO) published guidelines for antiviral prophylaxis in these patient populations. Here, we present an update of these guidelines for patients with solid and haematological malignancies undergoing antineoplastic treatment but not allogeneic stem cell transplantation. Relevant literature for reactivation of different viruses (herpes simplex virus (HSV), varicella zoster virus (VZV), hepatitis B virus (HBV) and respiratory viruses) is discussed to provide evidence-based recommendations for clinicians taking care of this patient population. We recommend a risk-adapted approach with (val)acyclovir against HSV and VZV in patients treated with alemtuzumab, bortezomib or purine analogues. Seasonal vaccination against influenza is recommended for all patients with solid or haematological malignancies regardless of antineoplastic therapy. Hepatitis B screening is recommended in lymphoproliferative disorders, acute leukaemia, and breast cancer, and during treatment with monoclonal anti-B-cell antibodies, anthracyclines, steroids and in autologous stem cell transplantation. In those with a history of hepatitis B prophylactic lamivudine, entecavir or nucleotide analogues as adefovir are recommended to prevent reactivation.

## Introduction

The risk of patients with solid tumours or haematological malignancies to contract viral infections is relatively low. Viral diseases occur most likely as reactivation of latent infections with herpes simplex virus (HSV), varicella zoster virus (VZV) and hepatitis B virus (HBV) being the most common viruses in these patients [1]. Apart from the setting of allogeneic stem cell transplantation, cytomegalovirus (CMV) and Epstein-Barr virus (EBV) play a subordinate role.

In recent years, the clinical relevance of viral infections of the respiratory tract has been increasingly recognized. Most viral infections are exogenous, primary infections. Influenza viruses are particularly important since patients with malignancies have an increased risk of contracting infections [2]. Moreover, an increased rate of secondary complications including bacterial pneumonia and fatal outcome has been observed [3].

The major risk factor for the occurrence of viral complications is the extent of cellular immunosuppression. The risk increases with the intensity and duration of T-cell suppression, as seen in the rate of viral complications during treatment with the T-cell antibody alemtuzumab. Severity and duration of neutropenia are of minor importance.

In 2006, the Infectious Diseases Working Party (AGIHO) of the German Society for Hematology and Medical Oncology (DGHO) published guidelines on antiviral prophylaxis in this patient population including recipients of allogeneic stem cell transplants [4]. The present aim of this guideline is to update the recommendations for patients with solid tumours and haematological malignancies. Recommendations for recipients of allogeneic stem cell transplants will be published separately and are not discussed here.

These guidelines have been prepared and composed by an expert panel from the AGIHO. Relevant literature published after 2006 was identified and reviewed using MEDLINE, CANCERLIT and the Cochrane library. Recent study results presented at major meetings in this field, including ASH, EHA, ASCO, ESMO, ECCMID or ICAAC, were additionally taken into account. The results were further discussed and finally approved by the assembly of the members of the AGIHO. The present article summarizes the development and rationale of the recommendations.

The aim of these guidelines is to provide physicians with evidence-based recommendations for the prevention of viral reactivations and primary viral infections in patients with solid tumours and haematological malignancies. In contrast to other published guidelines [5, 6], its relevance for a day-by-day use in the clinical setting is based on the evidence of recommendations as proposed by the Infectious Disease Society of America (IDSA) (see Table 1) [7]. The risk of viral complications and respective preventive strategies were determined depending on the underlying disease and specific therapies, i.e. chemotherapy with or without administration of monoclonal antibodies.

**Table 1:** Infectious Diseases Society of America—United States Public Health Service grading system for ranking recommendations

Category, grade	Definition
Strength of recommendation	
A	Good evidence to support a recommendation for use
B	Moderate evidence to support a recommendation for use
C	Poor evidence to support a recommendation
D	Moderate evidence to support a recommendation against use
E	Good evidence to support a recommendation against use
Quality of evidence	
I	Evidence from ≥1 properly randomized, controlled trial
II	Evidence from ≥1 well-designed clinical trial, without randomisation; from cohort or case-controlled analytic studies (preferably from >1 centre); from multiple time-series; or from dramatic results from uncontrolled experiments
III	Evidence from opinions of respected authorities, based on clinical experience, descriptive studies, or reports of expert committees

## Patient populations

### Conventionally dosed chemotherapy in solid tumours and haematological malignancies

Patients treated with conventionally dosed chemotherapy for their malignancy are at low risk for clinically relevant virus reactivations. The degree of cellular immunosuppression in patients with solid tumours is very limited. Furthermore, the majority of chemotherapeutics does not lead to a substantial suppression of T-cell function. Thus, the reactivation of HSV, VZV [8], EBV or CMV (30) constitutes a rare event in this patient population and does not require prophylaxis.

In contrast, the risk of primary viral infections of the upper respiratory tract is notably increased in patients with an active malignancy, with infections due to influenza, parainfluenza and respiratory syncytial virus (RSV) being the most clinically relevant [9–11]. As there are no effective prophylactic drugs, the instruction and performance of general hygiene measures represent an important prophylactic action [12, 13]. Despite the high variability in the response to the attenuated influenza vaccine in these patients [14], the influenza vaccine is recommended in patients with an active malignancy undergoing chemotherapy [15].

Induction and consolidation therapy in acute leukaemia patients results in a high risk for infectious complications. The occurrence of severe and persistent neutropenia with neutrophil counts below 500/μl or even 100/μl leads to an increased risk for febrile complications, mostly due to bacterial or fungal infections. Most viral infections during neutropenia are due to HSV [16]. A severe mucositis can impair the mucosal barrier leading to an increased risk for bacterial infections. Thus, the continuation of curative chemotherapy can be delayed. In addition, mucositis due to viruses results in significant deterioration of the patient’s general condition by causing severe pain, reduction of nutritional intake and cachexia. These complications are good reasons to prevent HSV reactivation. However, several studies could not prove a significant effect of acyclovir prophylaxis on the time and duration of antibiotic treatment, the number of febrile days, the rate of bloodstream infections and other opportunistic infections or mortality [17–19]. Accordingly, there is not enough evidence from randomized trials on antiviral prophylaxis for HSV in patients with acute leukaemia to establish a strong recommendation [20]. In contrast, the vaccination for influenza is recommended for all patients with acute leukaemia after intensive chemotherapy and for acute lymphoblastic leukaemia during the course of maintenance treatment [21].

Reactivation of hepatitis B virus infection is rather common in patients with solid tumours and haematological malignancies undergoing conventional chemotherapy and constitutes a serious complication for those patients [22–25]. In hepatitis B surface antigen (HBsAg) positive patients, the rate of reactivation is approximately 20–50 %, resulting in fulminant hepatitis and a high lethality in some cases. The risk is especially high in patients with malignant lymphoma and those undergoing treatment with anthracyclines [26, 27] or higher doses of steroids (10–20 mg prednisone daily or equivalent ≥4 weeks) [28, 29]. Primary antiviral prophylaxis with nucleoside analogues lamivudine and entecavir as well as nucleotide analogues adefovir and tenofovir are effective in the prevention of reactivation in patients with evidence of previous hepatitis B infection. Prophylaxis was associated with an 87 % relative risk reduction of reactivation [30] and also prevents fulminant hepatitis effectively [31]. Successful prophylaxis is of high prognostic value in patients with a curable underlying disease; patients can continue chemotherapy in time without interruption or dose reduction due to hepatitis.

Mono- and combination therapies with purine analogues like fludarabine or pentostatin result in a sustainable cellular immunosuppression and an increased risk for opportunistic infections [32, 33]. In the setting of first-line treatment, reactivations of VZV and HSV are rarely observed. Thus, primary prophylaxis with acyclovir cannot be recommended in this patient population.

In the presence of clinical risk factors including CD4+ cell count <50/μl, long-term treatment with steroids, persistent neutropenia, age more than 65 years or an advanced stage of underlying disease, antiviral prophylaxis can be reasonable [34]. Results from newer studies since 2006 do not substantially change the recommendations regarding risk and prophylactic strategy with acyclovir or valacyclovir to prevent VZV or HSV reactivation.

**Fig. 1:**
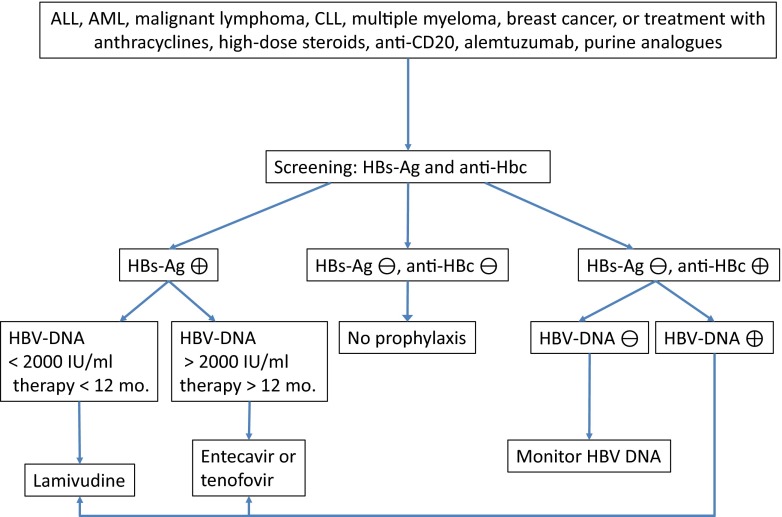
Algorithm for prophylaxis of HBV reactivation

In conclusion, no general recommendation regarding prophylactic medication for HSV, EBV or CMV in patients with solid tumours and haematological malignancies undergoing conventionally dosed chemotherapy can be given (see Table 2). The risk of viral reactivation is low due to only limited cellular immunosuppression, and evidence from randomized trials is missing. This is different for influenza and HBV; for these viruses, evidence-based strategies to prevent primary infection (influenza) or reactivation of latent infection (HBV) exist (see Table 2 and Fig. 1).

**Table 2:** Evidence-based recommendations for antiviral prophylaxis in patients with solid tumours and haematological malignancies except hepatitis B

	Chemotherapy	Rituximab	Alemtuzumab	Proteasome inhibitors	Purine analogues	Autologous SCT
HSV/VZV	None (CII)	None (CII)	Acyclovir (AII)	Acyclovir (AII)	Acyclovir^a^ (AII)	None (CII)
Influenza	Vaccination (AII)	Vaccination (BIII)	Vaccination (BIII)	Vaccination (AIII)	Vaccination (BIII)	Vaccination (BIII)
CMV	None (CII)	None (CII)	None (BII)	None (CIII)	None (CIII)	None (CII)
EBV	None (EIII)	None (EIII)	None (EIII)	None (EIII)	None (EIII)	None (EIII)
Resp. viruses adenovirus	None (CII)	None (CII)	None (CII)	None (CII)	None (CII)	None (CII)
HCV	None (CII)	None (CII)	None (CII)	None (CII)	None (CII)	None (CII)

^a^In the presence of risk factors: second-line therapy, prolonged treatment with steroids, CD4 count <50/μl, age >65 years, neutrophil count <1000/μ

### Monoclonal antibodies

Administration of monoclonal antibodies has become an integral part of treating malignancies. In the treatment of solid tumours, antibodies against growth factors of angiogenesis and against growth receptors like the EGF-receptor are well established. Examples include the combination of trastuzumab, bevacizumab or cetuximab with classical chemotherapy in the treatment of breast and colorectal cancer.

In lymphoproliferative diseases, targeted therapy against CD20 and—more recently—CD30 is widespread. The administration of rituximab, an anti-CD20 antibody, represents the basis of the treatment of B-cell lymphoma [35, 36]. Second generation antibodies like ofatumumab or obinutuzumab are about to be integrated in everyday clinical practice.

This treatment leads to a sustained B-cell depletion and, thus, increases the risk of viral reactivations. Most clinically relevant in this setting is the reactivation of HBV, which can occur in up to 50 % of patients with previous hepatitis B [37–40]. Reactivations of HSV, VZV, CMV, EBV and other viruses are more infrequent. The substantial cellular immunosuppression caused by the anti-CD20 therapy is also evident in associated cases of progressive multifocal leukencephalopathy as a result of JC virus reactivation [41].

On the basis of current evidence, there is no rationale for prophylaxis against HSV, VZV, EBV or CMV during B-cell antibody treatment. Nevertheless, particular attention should be paid to clinical signs of viral reactivation, as a wide variety of atypical pathogens can cause severe infections during the course of treatment. To prevent influenza infection, vaccination is recommended. Despite the predominantly inadequate immune response under treatment with B-cell antibodies, some results indicate reduced severity of influenza infections after vaccination [42, 43].

Additionally, all patients receiving B-cell antibodies should be screened for chronic hepatitis B as a clinical standard [44]. This recommendation has been expressed in guidelines of different societies [45–52]. The cost-effectiveness of this strategy has recently been demonstrated in patients receiving R-CHOP (rituximab plus cyclophosphamide, doxorubicin, vincristine and prednisone) for malignant lymphoma [53].

The algorithm of screening and antiviral prophylaxis for hepatitis B is explained in the chapter on antiviral substances and in Fig. 1.

The administration of anti-CD30 antibodies linked with a cytostatic (i.e. brentuximab vedotin) is an established alternative in the therapy of relapsed Hodgkin’s lymphoma and in CD30-positive anaplastic large-cell lymphoma [54]. There are reports of an increased incidence of opportunistic infections; viral reactivations, however, are not a major concern. On the basis of already existing data, no general recommendation on antiviral prophylaxis can be made.

Currently, alemtuzumab, an anti-CD52 antibody, is only rarely used for treatment of CLL. Several studies have shown that the incidence of reactivations of HSV, VZV and CMV is considerably high [55]. Therefore, prophylaxis with acyclovir for HSV and VZV as well as monitoring of CMV reactivation by PCR or detection of the early antigen in peripheral blood has become standard. No changes apply to the recommendations of 2006 [4].

### Proteasome inhibitors

Bortezomib is a proteasome inhibitor and a standard treatment for multiple myeloma. The substance is usually given in combination with dexamethasone or with classical cytostatics. It can be used both in primary therapy and for relapsed disease. Another molecule of this class is carfilzomib, a second-generation proteasome inhibitor, which has recently been approved by the Food and Drug Administration (FDA) [56].

The APEX study showed an increased VZV reactivation rate in patients receiving bortezomib [57]. These reactivations can be effectively prevented by low-dose acyclovir prophylaxis, as reported in several phase II trials and retrospective analyses [58–61]. However, to date, there are no randomized phase III trials addressing this question. On the basis of the given results, a recommendation for VZV prophylaxis by low-dose acyclovir or valacyclovir can be made. Further prophylactic agents are not indicated in this setting.

Patients with multiple myeloma present with marked immune deficiency that—as compared to other lymphoproliferative diseases—can be accompanied by a distinct antibody deficiency syndrome. Therefore, immunization is an important measure in preventing infections in this patient population [56]. Seasonal influenza vaccination is indicated for patients with multiple myeloma both with or without treatment with proteasome inhibitors [62].

### Autologous stem cell transplantation

The risk of opportunistic infections after autologous stem cell transplantation (SCT) correlates with the immune reconstitution. As T-cell depleted grafts are no longer used, the cellular immune deficiency is usually mild. However, it correlates with the extent of pre-treatment and the remission state of the underlying disease. Pre-treatment with high dose steroids or a transplantation after the second or higher relapse lead to marked cellular immunosuppression and thus to an increased risk for viral reactivations. Antiviral prophylaxis may thus be helpful in individual cases.

Reactivations of HSV and VZV leading to mucocutaneous infections are paramount after autologous SCT [63]. HSV infections may occur along with mucositis after high dose chemotherapy until the regeneration of the granulocytes. VZV reactivation is more frequent after reconstitution of granulopoesis until day 100 after transplantation. Reported incidences differ and vary from 8 to 20 % [64].

No new evidence-based data addressing this topic has been generated since 2006. In particular, no randomized trials on antiviral prophylaxis have been conducted. As a result, the recommendations of this guideline regarding antiviral prophylaxis after autologous SCT do not differ from those published previously [4]. General administration of acyclovir or valacyclovir for the prophylaxis of HSV or VZV reactivation is not recommended [20]. Such prophylaxis may depend on an individualized risk assessment per patient. Relevant factors for this include the extent of pre-treatment, the remission state at the time of transplantation, comorbidities, age, previous infections during the course of the disease and CD4+ count. However, no controlled randomized studies have been conducted to evaluate the efficacy of such an individualized approach.

The risk of CMV reactivation after autologous SCT without T-cell depletion is very low [65]. There is no rationale for prophylaxis.

Analogous to previously described clinical scenarios, there is an indication for HBV prophylaxis in patients with chronic hepatitis B. No changes were applied to the recommendations of 2006—including the recommendation for the seasonal influenza vaccination [21, 66].

### Small molecules and signal transduction inhibitors

The principle of signal transduction inhibition is an inherent part of modern tumour therapy today. Examples include mTOR inhibitors (e.g. everolimus) or interferences in the signal transduction of EGFR- or JAK2-pathways. A promising approach is seen in molecules, which interfere in the signal transduction of B-cell receptors (e.g. ibrutinib) and therefore present an attractive therapeutic option for B-CLL and B-cell lymphoma [67, 68]. Humoral as well as cellular immunosuppression may be common in patients treated with these agents, especially in those who are heavily pretreated. Therefore, antiviral prophylaxis may be indicated on an individual risk basis.

There are a growing number of reports on increased rates of opportunistic infections under this treatment [69]. However, in the COMFORT II trial, which compared the efficacy of ruxolitinib with the best available treatment in patients with myelfibrosis, the incidence of infections decreased over time without focus on reactivation of viral infections [70]. Wang et al. report similar results for a long-term follow-up of treatment with ibrutinib in patients with mantle-cell lymphoma [71]. Regarding reactivation of HBV, prophylaxis with lamivudine is an effective strategy to prevent reactivation [72].

An evidence-based recommendation for antiviral prophylaxis, thus, cannot be generated.

## Prophylactic treatment

### Herpes simplex virus and varicella zoster virus

The unaltered standard in the prophylaxis of HSV and VZV is acyclovir 400 mg tid or qid or valacyclovir 500 mg bid or tid [73]. Superiority of one of the two drugs could not yet be demonstrated [74, 75]. Treatment should be initiated during the first week of antineoplastic therapy and continued beyond the end of therapy. So far, the best duration of prophylaxis could not be determined. We recommend monitoring CD4+ T-cell levels and the continuation of antiviral prophylaxis until the count is above 200/μl. However, there are no randomized trials for this strategy.

Regarding prophylaxis of VZV during therapy with proteasome inhibitors, a lower dose of acyclovir or valacyclovir has shown to be effective. Dosages as low as 200–400 mg for acyclovir or 500 mg for valacyclovir daily or bid were successfully used [54–57]. Randomized trials comparing the different dosages, however, do not exist, and the risk of acyclovir resistance by using low doses has not been assessed yet.

### Influenza viruses

Seasonal vaccination against influenza can be recommended for all patients with solid tumours or haematological malignancies regardless of antineoplastic therapy [13, 21, 62, 76, 77]. Despite reduced immunological competence, there is evidence showing that 70–80 % of patients with malignancies exhibit seroconversion after a vaccination [78, 79].

Patients with malignant lymphoma or multiple myeloma show a reduced response in comparison to patients with solid tumours [80]. Hence, a second vaccination can be reasonable in this patient population. According to the results of the VACANCE trial, the rate of seroconversion in patients with solid tumours was increased from 44 to 73 % after a second administration of influenza vaccine [81]. Yet the optimal timing of a second vaccination—especially during ongoing tumour therapy—is unclear. Some results suggest a better effectiveness when administering the vaccination directly after chemotherapy instead of shortly before the next cycle [14].

In addition, vaccination of healthcare workers as well as family and household members is of particular importance to reduce influenza virus circulation and the risk of infection.

### Hepatitis B virus

The risk of HBV reactivation during chemo- or immunotherapy is sufficiently documented in the literature [82]. HBV reactivation is of clinical relevance due to the high associated morbidity and lethality [83]. Reactivations have been described in all patients with a history of hepatitis B (that is, anti-HBc positive) regardless of the serological constellation (presence or absence of HB-antigens or anti-HBs) [84].

The incidence of reactivation varies in different patient populations [24]. In patients with solid tumours, the reactivation rate of HBV was reported to be below 1 % [85]. Administration of anthracyclines is a negative prognostic factor in these patients [26, 27]. On the other hand, HBV reactivation occurs in 30–50 % of patients with malignant lymphoma [86], with even higher rates during treatment with rituximab [40, 44]

In conclusion, the following clinical risk factors are associated with an increased risk of HBV reactivation:

- Treatment with anthracyclines
- Treatment with steroids: >10–20 mg prednisone daily or equivalent for ≥4 weeks
- Treatment with monoclonal antibodies (rituximab, obinutuzumab, ofatumomab, alemtuzumab)
- Breast cancer as underlying disease
- Malignant lymphoma as underlying disease

Preemptive treatment with lamivudine, entecavir or nucleotide analogues as adefovir is standard in the prophylaxis of HBV reactivation [4, 45, 87]. This preemptive measure, however, requires the evidence of a previous infection. Therefore, patients with haematological malignancies or patients with solid tumours before planned tumour therapy should undergo a screening on HBV infection. To date, it remains unclear whether a general screening of all patients is needed.

We favour a risk-adapted approach and recommend HBV screening in patients with malignant lymphoma, multiple myeloma, CLL, AML, ALL and breast cancer, as well as patients planned for therapy with monoclonal B-cell antibodies, alemtuzumab, chemotherapy protocols based on the use of anthracyclines, higher doses of steroids and those undergoing autologous SCT (AII). In this patient population, tests for HBs-Ag and anti-HBc antibodies should be performed.

In case of HBsAg negativity and anti-HBc antibody negativity, antiviral prophylaxis is not indicated and immunisation should be considered. Patients with a negative HBsAg and a positive anti-HBc antibody test should be tested for HBV DNA. Those with a positive viral load should receive prophylaxis while those with a negative PCR should be screened for reactivation on a regular basis [45] (AIII). So far, the optimal approach in this risk group is not defined. Depending on the intensity of immunosuppression, monitoring should be repeated every 1–3 months. In patients who receive treatment with a particularly high risk of HBV reactivation (e.g. anti-CD20 targeted therapy), antiviral prophylaxis regardless of HBV-load should be considered.

In case of HBsAg positivity, the quantification of HBV DNA may determine the agent for antiviral prophylaxis (AI). Prophylactic treatment should be started together with the immunosuppressive therapy. While there are not randomized trials investigating the optimal duration of prophylaxis, reactivations have been described after the end of antineoplastic therapy. Thus, antiviral prophylaxis should be continued for 6–12 months after the completion of antineoplastic therapy (AII). The question of the most suitable antiviral drug for prophylaxis in this patient population is still unanswered. Most data on effectiveness exists for lamivudine. However, there are a few randomized trials with newer substances such as entecavir, adefovir or tenofovir [88]. Arguments for the latter include a higher antiviral potency and a lower risk of resistance development, which otherwise is substantially increasing after 12 months of treatment with lamivudine.

We suggest a risk-adapted approach in accordance with other recently published guidelines [30] (AIII). Prophylaxis with lamivudine is appropriate in short-term cancer treatment of approximately 4–6 months duration or in patients with a viral load below 2000 IU/ml, which is associated with a low risk of disease (42). In sustained immunosuppression for more than 12 months, i.e. in rituximab maintenance therapy, or in case of a high viral load above 2000 IU/ml, we recommend applying substances with a higher antiviral potency like entecavir or tenofovir (42) (see Fig. 1).

### Cytomegalovirus

In the above-described patient populations, there is no evidence for CMV prophylaxis. Serological monitoring of CMV reactivation by testing for early antigen or using PCR in peripheral blood is obligatory during treatment with alemtuzumab. It can be indicated in certain constellations with increased risk in patients undergoing autologous SCT. In principle, PCR is to be preferred due to its higher reliability not depending on the neutrophil count.

### Epstein-Barr virus

Reactivation of EBV is rare in this patient population. Prophylaxis is not recommended.

### Respiratory viruses—other than influenza virus and adenovirus

Patients with solid tumours or haematological malignancies have an increased risk for infections of the upper respiratory tract regardless of their tumour therapy. Usually these are primary infections. Therefore, patients should be monitored closely for clinical signs of infection. There is no indication for antiviral prophylaxis. However, patients with evidence of infection with respiratory viruses should be isolated to protect other patients from contracting the disease.

### Hepatitis C virus

Hepatitis C (HCV) infection does not constitute a contraindication for therapy in these patients including those undergoing autologous SCT. Standard cancer treatment in HCV positive patients yields similar disease-free survival and overall survival rates compared to those who are HCV negative [89]. However, we recommend consultation of a hepatologist while setting up the cancer treatment strategy. First, HCV infection is associated with increased hepatic toxicity during chemotherapy, and second, it might be appropriate to treat the viral infection simultaneously since the availability of interferon-free anti-HCV therapy [90]. But in conclusion, at this moment, no reasonable evidence-based preventive measure exists.

## Conclusions

Patients with solid tumours and haematological malignancies have—to different degrees—an increased risk for reactivation or primary acquisition of viral infections. The risk correlates with the intensity of cellular immunosuppression and, thus, with the type of therapy. Depending on this risk, evidence-based prophylaxis is indicated. This applies in particular to HBV reactivation, herpes zoster and primary infections with influenza virus.
